# Acute Change in Ventricular Contractility-Load Coupling After Corrective Surgery for Congenital Heart Defect: A Retrospective Cohort Study

**DOI:** 10.1007/s00246-019-02195-z

**Published:** 2019-09-03

**Authors:** Jimi Oh, In-Kyung Song, Junki Cho, Tae-Jin Yun, Chun Soo Park, Jae Moon Choi, Mijeung Gwak, Won-Jung Shin

**Affiliations:** 1 Department of Anesthesiology and Pain Medicine, Asan Medical Center, University of Ulsan College of Medicine, 88 Olympic-ro 43-gil, Songpa-gu, 05505 Seoul, Republic of Korea; 2 Department of Anesthesiology and Pain Medicine, Laboratory for Cardiovascular Dynamics, Asan Medical Center, University of Ulsan College of Medicine, 88 Olympic-ro 43-gil, Songpa-gu, 05505 Seoul, Republic of Korea; 3 Department of Pediatric Cardiac Surgery, Asan Medical Center, University of Ulsan College of Medicine, 88 Olympic-ro 43-gil, Songpa-gu, 05505 Seoul, Republic of Korea

**Keywords:** Cardiac surgical procedures, Echocardiography, Heart defects, Congenital, Pediatrics, Length of stay

## Abstract

Ventricular performance and its loading condition change drastically after surgical correction of congenital heart defect. Pressure–volume loops analysis can provide quantitative information about ventriculo-arterial coupling (VAC) indicating the interaction between ventricular contractility and loading condition. Therefore, we investigated changes in VAC after corrective surgery for ventricular septal defect (VSD)/tetralogy of Fallot (TOF), and implication of ventriculo-arterial decoupling as a prognostic factor of post-operative outcomes. In children with VSD/TOF, pre- and post-operative arterial elastance (Ea), end-systolic ventricular elastance (Ees) and VAC (Ea/Ees) were non-invasively estimated using echocardiographic parameters. Post-operative outcomes included maximum vasoactive-inotropic score, the duration of mechanical ventilation and hospital stay. Preoperatively, patients with VSD had significantly lower Ea and Ees than patients with TOF; however, VAC were preserved in both. In patients with VSD, post-operative Ea increased disproportionately to change in Ees, resulting in increased VAC. Post-operative higher VAC in patients with VSD was independently associated with maximum vasoactive-inotropic score (odds ratio [OR] 63.9; 95% Confidence Interval [CI] 4.0–553.0; *P* = 0.003), prolonged mechanical ventilation (OR 6.3; 95% CI 1.1–37.8; *P* = 0.044) and longer hospitalization (OR 17.6; 95% CI 1.6–187.0; *P* = 0.018). In patients with TOF, Ea and Ees reduced post-operatively; however, VAC remained unchanged and was not associated with post-operative outcomes. Despite of different loading condition, preoperative VAC maintained in both of VSD and TOF. However, particularly in VSD patients, abrupt increase in post-operative loading state induces contractility-load decoupling, which leads to worse post-operative outcomes.

**Electronic supplementary material:**

The online version of this article (10.1007/s00246-019-02195-z) contains supplementary material, which is available to authorized users.

## Introduction

In children with congenital heart defects (CHDs), intra- or extra-cardiac shunt is associated with the pressure or volume overloading of ventricles, which influences alterations in ventricular structures and mechanics [1]. The hemodynamic stabilization in such patients is ensured by changes in loading and ventricular structures as a compensatory mechanism. However, surgical correction of anatomical defects may lead to abrupt changes in loading status resulting in the deterioration of ventricular function. Therefore, it is crucial to assess ventricular function integrated with loading change, particularly during the immediate post-operative period.

A pressure–volume (P–V) relationship analysis is generally used to comprehensively assess ventricular performance during a cardiac cycle [2, 3]. Given the P–V loop, the left ventricular end-systolic elastance (Ees) can be estimated from end-systolic pressure (ESP) divided by end-systolic volume (ESV), reflecting the ventricular contractility [4]. Further, the arterial elastance (Ea) is calculated as ESP divided by stroke volume (SV), which represents the net arterial load [5]. The ratio of Ea to Ees is designated as ventriculo-arterial coupling (VAC) [6–8]. If Ees is effectively matched to Ea, the ventricle can be maximally efficient with favorable mechano-energetics [3, 9]. In children with CHDs, ESV and SV measured from echocardiography are influenced by the amount of shunt due to anatomical defects as well as effective systemic output. Therefore, Ea may be considered as a total afterload that works in the ventricles in these patients.

Senzaki et al. showed the characteristics of P–V relationship according to various loading conditions in specific types of CHDs [10, 11]. They found that, representatively, left ventricular preload is increased in ventricular septal defect (VSD), characterized by low Ees and Ea. Whereas preload is limited in significant pulmonary stenosis such as tetralogy of Fallot (TOF), which leads to small PV loop area and steep Ees slope [10]. However, it remains uncertain how parameters of P–V analysis change when anatomical shunts are surgically corrected and if coupling between ventricular contractility and load has prognostic significance.

In this study, we investigated the alterations in P–V relationship including Ees, Ea and VAC according to different loading conditions in children undergoing cardiac surgery for VSD and TOF, using non-invasive echocardiographic estimation. Additionally, we examined whether post-operative changes in Ees, Ea or VAC had any prognostic significance following corrective surgery.

## Materials and Methods

### Patients

This retrospective cohort study was approved by the Institutional Review Board and Institutional Ethics Committee of Asan Medical Center, Seoul, Korea in July 2017. The need for an informed consent from patients was waived by the Institutional Review Board. Electronic medical records of pediatric patients younger than 6 years with diagnoses of CHDs, who underwent cardiac surgery with cardiopulmonary bypass (CPB) between January 2011 and December 2016, were retrospectively reviewed. VSD and TOF having representative hemodynamic features of left ventricular volume overloading and underloading, respectively, were selected for the grouping of CHDs [10, 12]. Pediatric patients younger than 6 years, who were diagnosed with Kawasaki disease with normal echocardiographic findings during the same period, were assigned to the control group [10] and their medical records were reviewed. Exclusion criteria were as follows: pre-existing congenital or genetic anomaly, previous operations other than corrective surgery, complex heart disease and incomplete medical records.

### Clinical Variables and Post-operative Outcomes

Demographic variables included age, sex, weight, height, history of prematurity and birth weight. Intra-operative variables were anesthesia time, operation time, CPB time, aorta cross-clamp time and maximum vasoactive-inotropic score (VIS_max_) after weaning from CPB. Post-operative variables included the duration of stay in the intensive care unit, mechanical ventilation, post-operative VIS_max_ during the first 48 h and post-operative complications. The VIS was calculated as described by Gaies et al. [13].

### Echocardiographic Measurements

Two-dimensional transthoracic echocardiography (TTE) examination using a commercially available ultrasound system (Philips iE33, Philips Medical Systems, Andover, MA, USA) was performed by an experienced sonographer to determine the cardiac structures and function according to the American Society of Echocardiography recommendations [14], and attending pediatric cardiologists interpreted and confirmed results. TTE was performed preoperatively within 7 days before operation and before hospital discharge. Left ventricular end-diastolic volume (EDV), ESV, SV (EDV-ESV), ejection fraction (EF) and fractional shortening (FS) were measured using the Teichholz method or modified Simpson’s biplane method. All relevant data were normalized by each patient’s body surface area and designated as an index (ESVI, EDVI and SVI).

### Variables Derived from Left Ventricular P–V Relationships

Regarding arterial load, Ea was represented as the slope of ESP versus SVI in the PV loop [5]. To quantify ventricular contractility non-invasively, Ees was estimated as ESP divided by ESVI [4]. VAC was represented by the Ea/Ees ratio [6]. ESP was determined by non-invasive blood pressure measurement at the same time as TTE examination [5]. PV loop derived variables were summarized as follows:

$$ \begin{gathered} {\text{ESP }} = 0.{9} \times {\text{systolic blood pressure}} \hfill \\ {\text{Ea }} = {\text{ ESP}}/{\text{SVI}} \hfill \\ {\text{Ees }} = {\text{ ESP}}/{\text{ESVI}} \hfill \\ {\text{VAC }} = {\text{ Ea}}/{\text{Ees }} = {\text{ ESVI}}/{\text{SVI}} \hfill \\ \end{gathered} $$

### Statistical Analysis

All data were expressed as mean ± SD, median (IQR) or number (proportion) as appropriate. Missing data were managed either by the deletion of the case or variable. Categorical variables were analyzed using Pearson *χ*^2^ test or Fisher’s exact test, while continuous variables were analyzed using Student’s *t*-test or Mann–Whitney *U* test. One-way analysis of variance and Kruskal–Wallis test were applied to determine the differences between the groups, followed by a post-hoc Bonferroni test. The statistical significance of the change in the PV relationship before and after operation was determined using the paired *t*-test and Wilcoxon signed-rank test. Univariate logistic regression analysis was performed to determine the relationship between worse post-operative outcomes and PV loop variables. Worse post-operative outcomes were defined as post-operative VIS_max_ > 5, the duration of mechanical ventilation > 15 h and post-operative hospitalization > 7 days. Multivariable logistic regression analysis was performed, adjusting for the clinical variables that showed significant differences. For all analyses, a *P-*value of < 0.05 was considered statistically significant. All analyses were performed using Statistical Package for the Social Sciences software (IBM® SPSS® Statistics 23, SPSS Inc., IBM Corporation, Armonk, NY, USA).

## Results

**Fig. 1:**
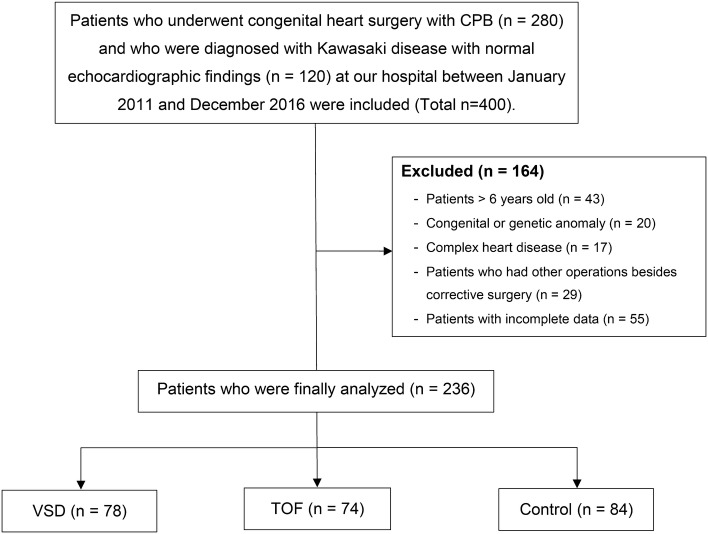
Flow chart of patient selection and classification. *CPB* cardiopulmonary bypass, *VSD* ventricular septal defect, *TOF* tetralogy of Fallot

Of the total 400 pediatric patients, whose electronic medical records were reviewed, 164 were excluded. Of the 236 patients included in the final analyses, 78 patients were assigned to the VSD group, 74 to the TOF group and 84 to the control group. The process of patient selection and classification is described in Fig. 1. The baseline demographic and clinical characteristics of pediatric patients with CHDs and controls are detailed in Table 1. In the post-operative period, permanent pacemaker implantation occurred in one patient and pneumonia in two patients in the VSD group. No patients died post-operatively during hospitalization.

**Table 1:** Demographic and clinical characteristics of pediatric patients undergoing congenital heart surgery

	VSD (*n* = 78)	TOF (*n* = 74)	Control (*n* = 84)
Demographic variables			
Age (month)	12.0 [3.0–23.3]*	6.00 [4.00–8.00]*^†^	23.0 [10.3–34.8]
Sex, male	54 (69)	57 (58)	49 (58)
Weight (kg)	9.45 [5.68–12.0]*	6.50 [5.70–7.78]*^†^	12.2 [9.13–14.5]
Height (cm)	76.3 [60.0–89.7]*	63.2 [59.5–67.8]*^†^	88.8 [72.5–97.3]
Body surface area (m^2^)	0.43 [0.29–0.53]	0.32 [0.29–0.36]*^†^	0.54 [0.41–0.62]
Birth weight (kg)	3.13 [2.71–3.40]	3.03 [2.65–3.46]	
Prematurity	6 (8)	12 (12)	
Intra-operative variables			
Anesthesia time (min)	248 ± 32	285 ± 51^†^	
Operation time (min)	196 ± 32	232 ± 53^†^	
CPB time (min)	69.8 ± 22	114 ± 31^†^	
Aorta cross-clamp time (min)	42.7 ± 15	67.4 ± 20^†^	
VIS_max_ after weaning from CPB	5.00 [4.50–5.00]	5.00 [4.00–5.00]	
Post-operative variables			
Duration of MV (h)	11.2 [4.00–15.3]	13.0 [11.0–16.0]^†^	
VIS_max_ during post-operative 48 h	3.00 [0.00–3.81]	3.00 [0.00–6.80]^†^	
Post-operative hospital stay	6 [5–7]	7 [6–8]	

Values are mean ± SD, median [IQR], or number (proportion)

*VSD* ventricular septal defect, *TOF* tetralogy of Fallot, *CPB* cardiopulmonary bypass, *VIS*_*max*_ maximum vasoactive-inotropic score, *MV* mechanical ventilation

*P < 0.05 vs. Control group, ^†^*P* < 0.05 vs. VSD group

### Changes in Left Ventricular P–V Relationship

**Fig. 2:**
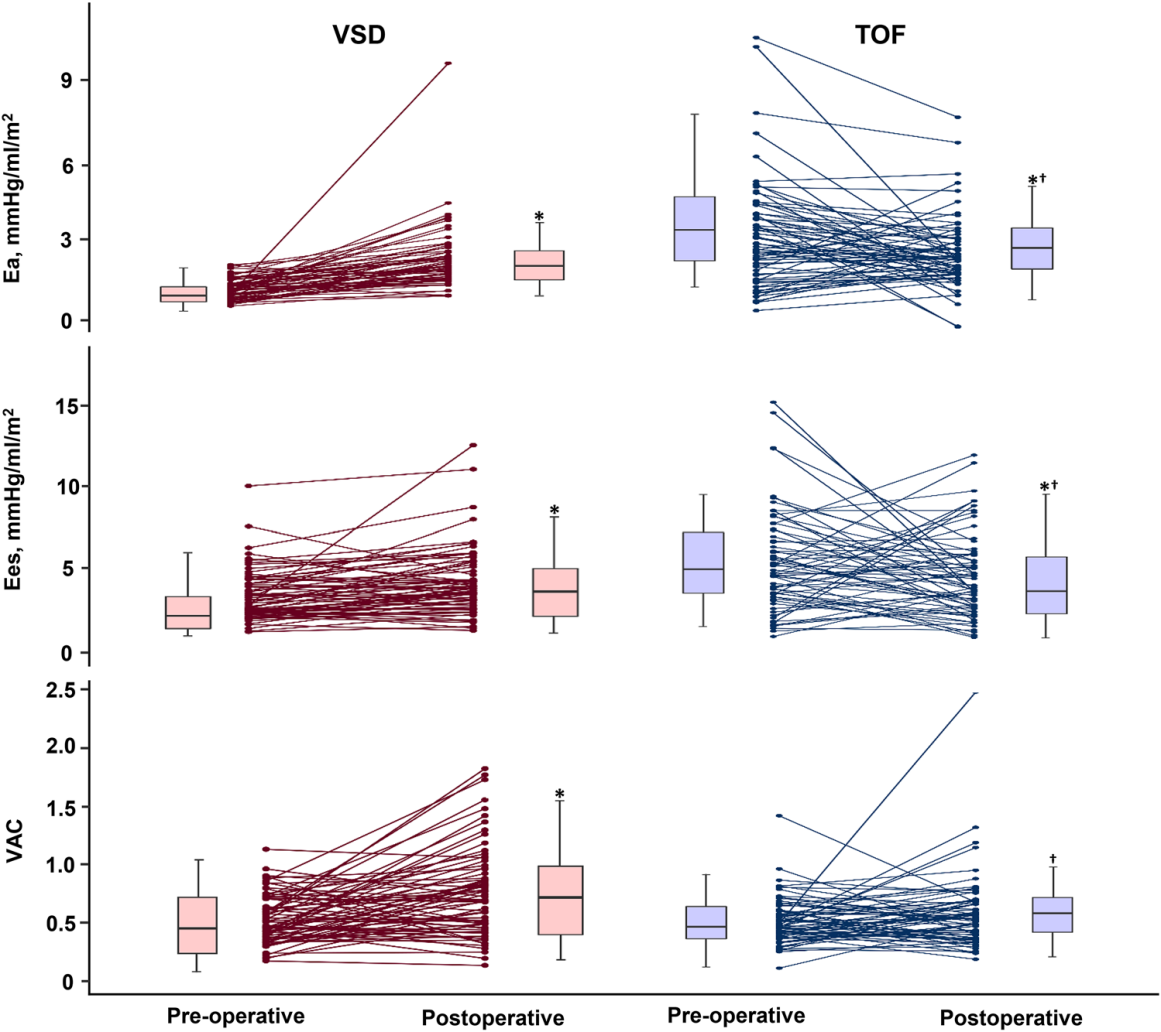
Individual changes in arterial elastance (Ea), end-systolic ventricular elastance (Ees) and ventriculo-arterial coupling (VAC) according to the type of congenital defects, pre- and post- operation. Post-operative Ea, Ees and VAC were significantly higher than pre-operative Ea, Ees and VAC in the ventricular septal defect (VSD) group. Contrary to patients with VSD, the Ea and Ees were reduced post-operation in the tetralogy of Fallot group. However, VAC did not significantly change. *VSD* ventricular septal defect, *TOF* tetralogy of Fallot. *P < 0.05 vs. pre-operative data; ^†^*P* < 0.05 vs. VSD group

**Fig. 3:**
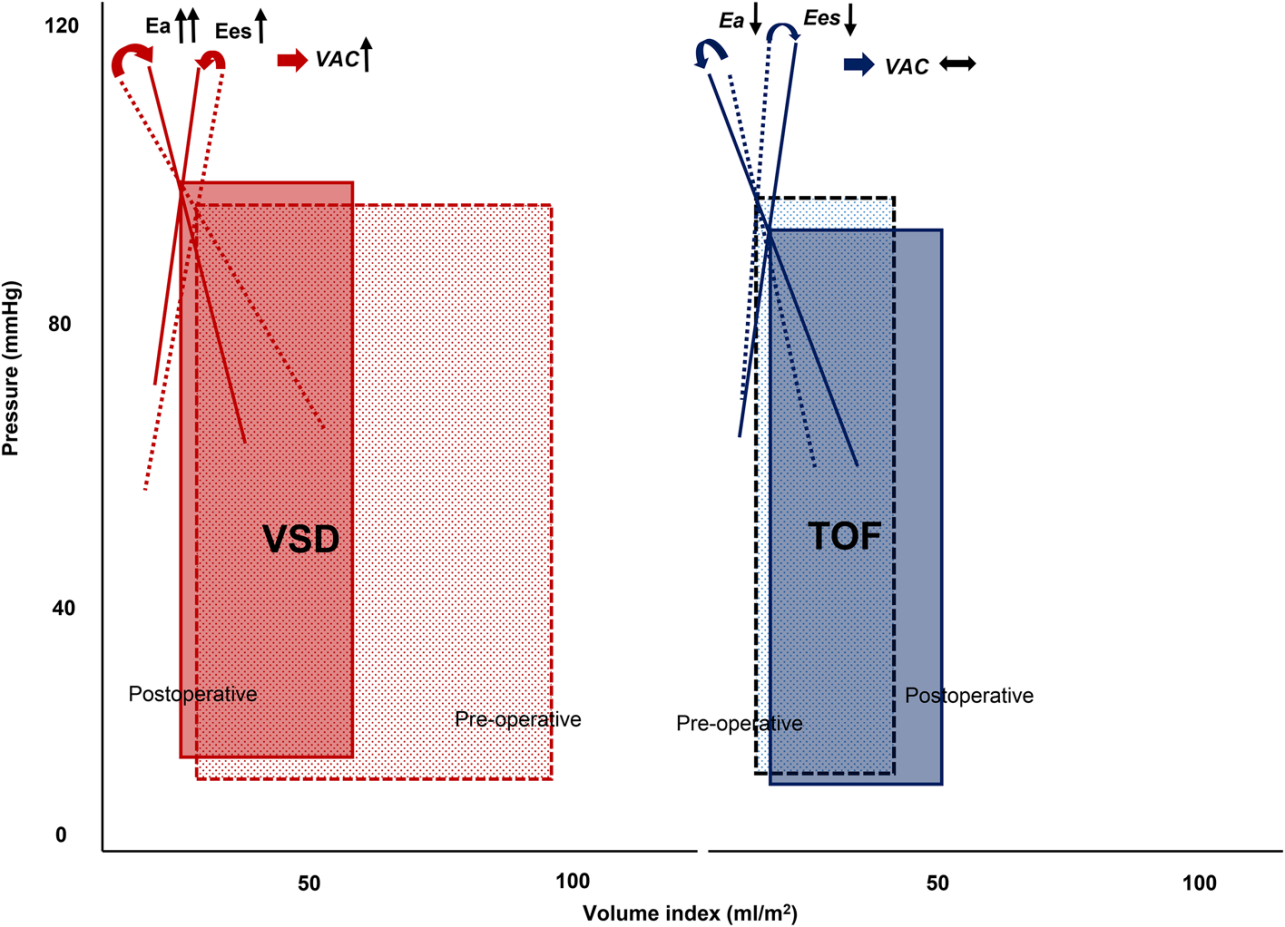
Example of pressure–volume loops. Compared with pre-operative patients with ventricular septal defect (VSD) (red-dashed lines), post-operative patients with VSD show a significant increase in arterial elastance (Ea) due to a sudden decrease in stroke volume, resulting in disproportionate changes in Ea and end-systolic ventricular elastance (Ees). Consequently, ventriculo-arterial coupling (VAC) increases on a left-shifted pressure–volume loop (red lines). However, compared with pre-operative patients with tetralogy of Fallot (TOF) (blue dashed lines), post-operative patients with TOF have decreased Ea and Ees, whereas VAC shows no change on a right-shifted pressure–volume loop (blue lines). *VSD* ventricular septal defect, *TOF* tetralogy of Fallot, *Ea* arterial elastance, *Ees* end-systolic ventricular elastance, *VAC* ventriculo-arterial coupling

In patients with VSD, pre-operative EDVI, ESVI and SVI were higher compared to those in controls and in patients with TOF. Patients with VSD also had lower Ea (1.49 ± 0.42 mmHg/ml/m^2^) compared to controls (2.08 ± 0.56 mmHg/ml/m^2^, *P* < 0.001) (Table 2). Nevertheless, VAC was maintained (0.48 ± 0.14 vs. 0.52 ± 0.13, *P* = 0.060) because of low basal Ea, which was matched with decreased Ees. After surgical correction, EDVI (− 31%) notably reduced compared to the reduction in ESVI (− 20%); thus, SVI was reduced. Post-operative ESP was not different from pre-operative ESP; therefore, Ea markedly increased (from 1.49 ± 0.42 to 2.54 ± 1.20 mmHg/ml/m^2^, *P* < 0.001), coinciding with a rise in VAC (from 0.48 ± 0.14 to 0.66 ± 0.30, *P* < 0.001) despite an increase in Ees (from 3.40 ± 1.49 to 4.38 ± 2.23 mmHg/ml/m^2^, *P* < 0.001), on the left-shifted PV loop (Figs. 2 and 3).

**Table 2:** Perioperative transthoracic echocardiographic measurements of study population

	VSD (*n* = 78)	TOF (*n* = 74)	Control (*n* = 84)
Pre-operative data			
ESP (mmHg)	88.7 ± 10.3	87.2 ± 12.3	90.2 ± 15.5
EDVI (ml/m^2^)	93.2 ± 23.4*	43.3 ± 23.5*^†^	68.4 ± 14.9
ESVI (ml/m^2^)	29.8 ± 10.2*	13.0 ± 8.21*^†^	23.3 ± 7.51
SVI (ml/m^2^)	63.4 ± 16.1*	30.3 ± 16.9*^†^	45.1 ± 9.32
EF (%)	68.2 ± 6.40	70.1 ± 8.60*	66.3 ± 6.01
FS (%)	37.1 ± 5.10	37.9 ± 7.63*	35.5 ± 4.62
Ea (mmHg/ml/m^2^)	1.49 ± 0.42*	3.70 ± 2.03*^†^	2.08 ± 0.56
Ees, (mmHg/ml/m^2^)	3.40 ± 1.49	10.3 ± 10.4*^†^	4.23 ± 1.51
VAC	0.48 ± 0.14	0.45 ± 0.18*	0.52 ± 0.13
Post-operative data			
ESP (mmHg)	88.7 ± 7.82	82.2 ± 6.33^†§^	
EDVI (ml/m^2^)	63.9 ± 19.6^§^	52.4 ± 48.6^†^	
ESVI (ml/m^2^)	24.2 ± 10.6^§^	17.4 ± 16.7^†§^	
SVI (ml/m^2^)	39.7 ± 13.3^§^	34.9 ± 33.0	
EF (%)	62.4 ± 10.0^§^	67.5 ± 10.0^†^	
FS (%)	32.7 ± 7.23^§^	36.0 ± 7.52^†^	
Ea (mmHg/ml/m^2^)	2.54 ± 1.20^§^	3.05 ± 1.32^†§^	
Ees (mmHg/ml/m^2^)	4.38 ± 2.23^§^	6.82 ± 3.60^†§^	
VAC	0.66 ± 0.30^§^	0.51 ± 0.21^†^	

Data are presented as mean ± SD

*VSD* ventricular septal defect*, TOF* tetralogy of Fallot, *ESP* end-systolic pressure; *EDVI* end-diastolic volume index, *ESVI* End-systolic Volume Index, *SVI* Stroke Volume Index, *EF* ejection fraction; *FS* fractional shortening; *Ea* arterial elastance, *Ees*, end-systolic elastance, *VAC* ventriculo-arterial coupling

*P < 0.05 vs. Control group, ^†^*P* < 0.05 vs. VSD group, ^§^*P* < 0.05 vs. Pre-operative data

In patients with TOF, pre-operative EDVI, ESVI and SVI were significantly lower than those in controls and in patients with VSD (Table 2). Patients with TOF had markedly higher Ees (10.30 ± 10.41 mmHg/ml/m^2^) compared with controls (4.23 ± 1.51 mmHg/ml/m^2^, *P* < 0.001) and patients with VSD (3.40 ± 1.49 mmHg/ml/m^2^, *P* < 0.001). Ea (3.70 ± 2.03 mmHg/ml/m^2^) also elevated significantly compared with the other groups (Controls, 2.08 ± 0.56 mmHg/ml/m^2^, *P* < 0.001 and VSD, 0.49 ± 0.42 mmHg/ml/m^2^, *P* < 0.001). As a result, the represented VAC was slightly lower than that of controls (0.45 ± 0.18 vs. 0.52 ± 0.13, *P* = 0.005, respectively). Contrary to patients with VSD, Ea and Ees reduced after surgery (3.05 ± 1.32 mmHg/ml/m^2^, *P* = 0.012 and 6.82 ± 3.60 mmHg/ml/m^2^, *P* = 0.019, respectively). Post-operative PV loops were slightly shifted to the right (Fig. 3); however, VAC did not significantly change (0.51 ± 0.21, *P* = 0.059).

### Association Between P–V Loop-Derived Variables and Post-operative Outcomes

In patients with VSD, post-operative Ees indicating ventricular contractility was significantly associated with high VIS_max_ (>5) and prolonged hospital stay (>7 post-operative days) on univariate analysis; however, it was not significant after multivariate adjustment. A post-operative increase in VAC in patients with VSD was independently associated with high VIS_max_ (odds ratio [OR] 63.9; 95% Confidence Interval [CI] 4.02–553.0; *P* = 0.003), prolonged duration of mechanical ventilation (>15 h) (OR 6.31; 95% CI 1.05–37.8; *P* = 0.044) and longer post-operative hospital stay (OR 17.6; 95% CI 1.64–187.0; *P* = 0.018) (Table 3). The other clinical variable associated with poor post-operative outcomes was CPB time (OR 1.02; 95% CI 1.00–1.04; *P* = 0.045) (see Supplemental Digital Content 1, which provides data on risk factor analyses). In patients with TOF, in contrast, post-operative changes in PV loop-derived variables including VAC were not associated with post-operative outcomes.

**Table 3:** Univariate and multivariate analyses of risk factors for poor post-operative outcome after congenital heart surgery

	VSD				TOF			
	Univariate		Multivariate		Univariate		Multivariate	
	OR (95% CI)	*P*	OR (95% CI)	*P*	OR (95% CI)	*P*	OR (95% CI)	*P*
Post-operative VIS_max_ > 5								
Ea	0.97 (0.55–1.70)	0.908			0.87 (0.56–1.35)	0.869		
Ees	0.40 (0.20–0.77)	**0.007**	0.58 (0.25–1.33)	0.198	0.91 (0.77–1.08)	0.273		
VAC	38.0 (3.58–402)	**0.003**	63.9 (4.02– 553)	**0.003**	0.88 (0.07–11.1)	0.923		
Duration of mechanical ventilation > 15 h								
Ea	1.34 (0.87–2.06)	0.189			1.02 (0.69–1.51)	0.924		
Ees	0.90 (0.69–1.19)	0.470			1.06 (0.92–1.22)	0.441		
VAC	6.98 (1.20–40.6)	**0.031**	6.31 (1.05–37.8)	**0.044**	0.31 (0.02–4.33)	0.382		
Post-operative hospital stay > 7 days								
Ea	1.00 (0.61–1.65)	0.988			1.14 (0.77–1.67)	0.521		
Ees	0.57 (0.35–0.94)	**0.027**	0.75 (0.38–1.51)	0.420	1.03 (0.90–1.19)	0.653		
VAC	11.0 (1.56–77.0)	**0.016**	17.6 (1.64–187)	**0.018**	0.94 (0.08–10.6)	0.944		

Data are OR (95% CI)

Bold values indicate *P* < 0.05

*OR* odds ratio, *CI* Confidence Interval, *VSD* ventricular septal defect, *TOF* tetralogy of Fallot, *VIS*_*max*_ maximum vasoactive-inotropic score, *Ea* arterial elastance, *Ees* end-systolic elastance, *VAC* ventriculo-arterial coupling

## Discussion

In this study, we investigated the characteristics of the P–V relationship estimated non-invasively from echocardiographic measurements before and after surgery for VSD and TOF with different loading conditions.VSD patients represented low-basal Ea and Ees and post-operative Ea increased in disproportion to the change of Ees. As a result, relationship between ventricular contractility and loading was uncoupled on left-shifted P–V loops immediately after surgery. Accordingly, VAC in children with VSD was elevated by abrupt changes in loading, which was associated with worse post-operative outcomes. Meanwhile, TOF patients characterized by high-basal Ea and Ees, and post-operative VAC were still maintained and not related to worse outcomes.

Basal interaction between ventricular contractility and loading are matched within a narrow range to optimize cardiac energetic efficiency [6]. In our results, baseline VAC was maintained in patients with VSD and TOF, which were similar to the controls. These results suggest that the ventriculo-loading system can allow to maximize energy efficiency by compensatory mechanisms, such as volume-overloading or -underloading. Previously, Senzaki et al. demonstrated that basal characteristics of the P–V relationship showed obvious distinction according to the type and severity of congenital heart disease in children [10]. As expected, in patients with VSD with left-to-right shunt, increase in EDVI is caused by recirculation through the pulmonary system inducing the left ventricular volume overloading [15]. Systemic outflow can be maintained with increased EDVI, despite a slight increase in ESVI at the same time. To overcome the significantly overloaded volumes, the left ventricle is remodeled into a dilated chamber with a thinning of the myocardium, which caused impaired ventricular function [1]. Additionally, the afterload against the left ventricle is reduced by shunting blood towards the low-resistance pulmonary system. In turn, reduced afterload causes the increased SVI and decreased Ea. Consequently, a decline in ventricular performance was masked by a significant reduction of Ea, shown by Ees within normal limits.

After surgical correction for VSD, the P–V relationship could be changed abruptly. Left-to-right shunt is disappeared, and volume overload is relieved leading to an instant reduction in EDVI. However, ESVI slightly decreases or remains stationary following the block of the shunt because the impaired ventricle cannot readily restore the systolic function [16]. Regarding change of the P–V relationship, Ea, which is the entire loading upon the ventricle as well as net arterial load, is elevated relatively higher than the change in Ees. In turn, VAC can be frequently disrupted with the disparity in Ees and Ea. It has been demonstrated that higher VAC reflects significantly compromised and less efficient cardiovascular performance in children with cardiac disease and heart failure [17–19]. We first revealed that an increase in VAC in patients with VSD was associated with post-operative outcomes. These results highlight that it is important to comprehensively assess the integration of LV contractility and loading condition using the P–V relationships during the critical post-operative period. Furthermore, in patients with VSD, the evaluation of cardiovascular performance including the ventricular contractility, vascular tone, and its interaction will enable individualized perioperative management. Based on the quantitative assessment of loading condition and LV contractility, personalized and tailored treatment could be applied to optimize VAC and improve post-operative outcomes.

On the contrary, TOF is characterized by significantly decreased EDVI and ESVI [12, 20]. Although obstruction of the right ventricular outflow directly impacts the right ventricular mechanics, the left ventricle is also influenced by persistent exposure to significantly limited preload and excessive afterload. As a result, the ventricle is adapted to volume-underloading and pressure overloading by maximizing stroke volume on the steep end-systolic P–V relationship [10]. Ultimately, VAC can be preserved with the rise in Ea and Ees.

After total correction of TOF, there were no significant differences in left ventricular volumes, and VAC was also maintained on the similar P–V loop with decreases in Ees coinciding with reduced Ea. We found that the change in VAC could not predict post-operative outcomes after the repair of TOF. Because pulmonary stenosis burdens the right ventricle rather than the left ventricle, right ventricular dysfunction has generally been targeted for management after surgery for TOF [21, 22]. Clinical implications of late post-operative cardiac derangement in TOF have also been focused on the right ventricle-pulmonary artery relationship [23]. For this reason, alteration in the left ventricular mechanics according to loading changes may have limitations in reflecting post-operative prognosis. However, it has been shown that the left ventricular performance is influenced by negative inter-ventricular interactions because increased right chamber size impedes left function and volume [20, 24]. To that regard, it is also crucial to assess the performance of the left ventricle as the systemic ventricle during the early post-operative period with sudden loading change. Our study is of high value in that we investigated alterations in the left ventricular P–V relationship before and immediately after surgical correction in TOF.

In this study, Ees was obtained on the end-systolic P–V relationship using a simple equation: Ees = ESP/ESVI [25], which requires only echocardiographic ventricular volume and non-invasive blood pressure. A previous study validated several methods for non-invasive estimation of Ees in children [26]. Unlike adults, the single-beat estimation of Ees using Doppler time intervals is overestimated in children, whose heart rates are significantly higher than adults to allow accurate calculation of time intervals. Consequently, the ratio of ESP to ESVI shows the best agreement with invasive measurement [26]. However, because echocardiographic measurement of ventricular volumes may be unreliable if morphological defects exist, it remains unclear if this method can be applied in children who have congenital heart disease with large ventricular defects. Nevertheless, using non-invasive method, we can easily evaluate the changes in LV contractility and loading condition in a short time.

## Study Limitations

There are several limitations to this study. First, this single-center study was performed as a retrospective analysis, reviewing routine pre- and post-operative evaluations. Therefore, these results should be interpreted with caution because the timing of the echocardiographic evaluation could not be rigorously controlled. Moreover, post-operative outcomes were relatively favorable, such as median VIS_max_ of 3 and hospital stay of 6 and 7 days, given the small size of the population. Although we adjusted for confounding factors that affected post-operative outcomes, further study in larger population is required to clarify the prognostic impact of VAC on the clinical outcomes and to discriminate the cut-off value with narrow CIs. Second, this simple method is to assume that the volume intercept of the P–V curve (V_0_) is zero [25]. Therefore, VAC may have discrepancy from invasive analysis of P–V relations by taking V_0_, especially in VSD patients who have increased ESVI. It has been reported that values of area-axis intercept are not significantly different from healthy controls, using pressure–area analysis constructed by ventricular area measurement [10]. However, there is still little known about how accurate VAC is estimated by non-invasive single-beat method promising in children with CHD, therefore, our results should be interpreted with caution when applied to various types of CHD. Third, as mentioned above, echocardiographic indices may be problematic in CHDs with abnormal left ventricular geometry. Senzaki et al. validated the feasibility of ventricular-loading dynamics using pressure–area analysis constructed by ventricular area measurement [10]. Instantaneous quantification of VAC by pressure–area analysis may provide useful information for management, particularly during the critical post-operative period.

## Conclusions

Preoperative variables derived from P–V loops can reflect baseline characteristics of ventricular performance-integrated loading condition, according to volume loading status of CHDs. In the VSD and TOF groups, VAC was favorably maintained, suggesting that ventricular contractility may be matched with loading abnormalities to preserve systemic outflow. However, abrupt post-operative changes of loading on the left ventricle may induce contractility-load decoupling. Especially in patients with VSD, an impaired ventricular function is unmasked by the disproportionate change in loading resulting in an increased VAC that is associated with worse post-operative outcomes. Therefore, our results suggest that non-invasive and simple estimation of VAC by P–V analysis helps in determining the appropriate target to optimize treatment after surgical correction for CHDs.

## Electronic supplementary material

Below is the link to the electronic supplementary material.

Supplementary file1 (DOCX 42 kb)

## Abbreviations

VSD: Ventricular septal defect
TOF: Tetralogy of Fallot
CHDs: Congenital heart defects
CPB: Cardiopulmonary bypass
P–V loop: Pressure–volume loop
Ea: Arterial elastance
Ees: End-systolic ventricular elastance
VAC: Ventriculo-arterial coupling
LVEDV: Left ventricular end-diastolic volume
LVESV: Left ventricular end-systolic volume
EF: Ejection fraction
FS: Fractional shortening
VIS_max_: Maximum vasoactive-inotropic score
