# Supplementary material for: Acute Change in Ventricular Contractility-Load Coupling After Corrective Surgery for Congenital Heart Defect: A Retrospective Cohort Study
Source: Pediatr Cardiol. 2019 Sep 3;40(8):1618–26. doi: 10.1007/s00246-019-02195-z (PMC6848039; doi:10.1007/s00246-019-02195-z)
Supplement: Supplementary file 1 — Supplementary file1 (DOCX 42 kb) [file 246_2019_2195_MOESM1_ESM.docx]

**Supplemental Digital Content 1**

Univariate and multivariable analyses of risk factors for post-operative vasoactive-inotropic score and duration of mechanical ventilation after congenital heart surgery.

|  | **VSD** | | | | **TOF** | | | |
| --- | --- | --- | --- | --- | --- | --- | --- | --- |
|  | Univariate | | Multivariable | | Univariate | | Multivariable | |
|  | OR (95% CI) | *P* | OR (95% CI) | *P* | OR (95% CI) | *P* | OR (95% CI) | *P* |
| *Post-operative VIS_max_ >5* | | | | | | | | |
| Ea | 0.97 (0.55–1.70) | 0.908 |  |  | 0.87 (0.56–1.35) | 0.869 |  |  |
| Ees | 0.40 (0.20–0.77) | 0.007*^*^* | 0.58 (0.25–1.33) | 0.198 | 0.91 (0.77–1.08) | 0.273 |  |  |
| VAC | 38.0 (3.58–402) | 0.003 *^*^* | 63.9 (4.02–553) | 0.003 *^*^* | 0.88 (0.07–11.1) | 0.923 |  |  |
| Age | 0.99 (0.95–1.04) | 0.682 |  |  | 1.12 (1.00–1.24) | 0.045 |  |  |
| Body weight | 0.65 (0.48–0.88) | 0.006 *^*^* | 0.99 (0.99–1.00) | 0.358 | 1.02 (0.76–1.36) | 0.916 |  |  |
| CPB time | 1.05 (1.02–1.09) | 0.003 *^*^* | 1.07 (1.02–1.12) | 0.003 *^*^* | 1.02 (1.00–1.04) | 0.045 *^*^* | 1.02 (1.00–1.04) | 0.045 *^*^* |
| Prematurity | 0.00 (0.00–0.00) | 0.999 |  |  | 0.60 (0.06–5.71) | 0.657 |  |  |
| Ejection Fraction | 0.97 (0.88–1.08) | 0.603 |  |  | 1.02 (0.97–1.09) | 0.425 |  |  |
| *Duration of mechanical ventilation >15 hours* | | | | | | | | |
| Ea | 1.34 (0.87–2.06) | 0.189 |  |  | 1.02 (0.69–1.51) | 0.924 |  |  |
| Ees | 0.90 (0.69–1.19) | 0.470 |  |  | 1.06 (0.92–1.22) | 0.441 |  |  |
| VAC | 6.98 (1.20–40.6) | 0.031 *^*^* | 6.31 (1.05–37.8) | 0.044 *^*^* | 0.31 (0.02–4.33) | 0.382 |  |  |
| Age | 1.01 (0.98–1.04) | 0.601 |  |  | 1.05 (0.96–1.15) | 0.275 |  |  |
| Body weight | 1.00 (0.99–1.00) | 0.391 |  |  | 0.99 (0.99–1.00) | 0.181 |  |  |
| CPB time | 1.03 (1.01–1.06) | 0.020 *^*^* | 1.03 (1.00–1.06) | 0.028 *^*^* | 1.02 (1.00–1.04) | 0.056 |  |  |
| Prematurity | 0.60 (0.07–5.48) | 0.651 |  |  | 1.39 (0.22–8.95) | 0.726 |  |  |
| Ejection Fraction | 0.94 (0.97–1.02) | 0.150 |  |  | 0.96 (0.90–1.02) | 0.161 |  |  |
| *Post-operative hospital stay >7 days* | | | | | | | | |
| Ea | 1.00 (0.61–1.65) | 0.988 |  |  | 1.14 (0.77–1.67) | 0.521 |  |  |
| Ees | 0.57 (0.35–0.94) | 0.027 *^*^* | 0.75 (0.38–1.51) | 0.420 | 1.03 (0.90–1.19) | 0.653 |  |  |
| VAC | 11.0 (1.56–77.0) | 0.016 *^*^* | 17.6 (1.64–187) | 0.018 *^*^* | 0.94 (0.08–10.6) | 0.944 |  |  |
| Age | 1.01 (0.97–1.04) | 0.810 |  |  | 1.00 (0.91–1.09) | 0.987 |  |  |
| Body weight | 0.81 (0.67–0.98) | 0.033 *^*^* | 1.00 (0.99–1.00) | 0.698 | 0.79 (0.58–1.07) | 0.123 |  |  |
| CPB time | 1.08 (1.03–1.12) | 0.001 *^*^* | 1.08 (1.04–1.13) | 0.001 *^*^* | 1.01 (0.99–1.03) | 0.347 |  |  |
| Prematurity | 1.00 (0.11–9.34) | 1.000 |  |  | 8.95 (0.94–85.0) | 0.056 |  |  |
| Ejection Fraction | 0.97 (0.88–1.06) | 0.507 |  |  | 0.96 (0.91–1.02) | 0.222 |  |  |

Data are OR (95% CI). *^*^* *P* < 0.05. OR, odds ratio; CI, Confidence Interval; VSD, ventricular septal defect; TOF, tetralogy of Fallot; O.R., odds ratio; CI, confidence interval; VIS_max_, maximum vasoactive-inotropic score; Ea, arterial elastance; Ees, end-systolic elastance; VAC, ventriculo-arterial coupling; CPB, cardiopulmonary bypass. Multivariable, adjusted by backward regression of perioperative and pressure-volume loop related variables; Ea, Ees, VAC, age, body weight, body weight, prematurity, EF.
